# Optimization of ATAC-seq in wheat seedling roots using INTACT-isolated nuclei

**DOI:** 10.1186/s12870-023-04281-0

**Published:** 2023-05-22

**Authors:** Juan M. Debernardi, German Burguener, Kerry Bubb, Qiujie Liu, Christine Queitsch, Jorge Dubcovsky

**Affiliations:** 1 University of California, Davis, CA 95616 USA; 2 Howard Hughes Medical Institute, Chevy Chase, MD 20815 USA; 3 Dept. of Biology, University of Washington, Seattle, WA 98195 USA

**Keywords:** Wheat, Open chromatin, Regulatory regions, ATAC-seq, INTACT

## Abstract

**Background:**

The genetic information contained in the genome of an organism is organized in genes and regulatory elements that control gene expression. The genomes of multiple plants species have already been sequenced and the gene repertory have been annotated, however, *cis*-regulatory elements remain less characterized, limiting our understanding of genome functionality. These elements act as open platforms for recruiting both positive- and negative-acting transcription factors, and as such, chromatin accessibility is an important signature for their identification.

**Results:**

In this work we developed a transgenic INTACT [isolation of nuclei tagged in specific cell types] system in tetraploid wheat for nuclei purifications. Then, we combined the INTACT system together with the assay for transposase-accessible chromatin with sequencing [ATAC-seq] to identify open chromatin regions in wheat root tip samples. Our ATAC-seq results showed a large enrichment of open chromatin regions in intergenic and promoter regions, which is expected for regulatory elements and that is similar to ATAC-seq results obtained in other plant species. In addition, root ATAC-seq peaks showed a significant overlap with a previously published ATAC-seq data from wheat leaf protoplast, indicating a high reproducibility between the two experiments and a large overlap between open chromatin regions in root and leaf tissues. Importantly, we observed overlap between ATAC-seq peaks and *cis*-regulatory elements that have been functionally validated in wheat, and a good correlation between normalized accessibility and gene expression levels.

**Conclusions:**

We have developed and validated an INTACT system in tetraploid wheat that allows rapid and high-quality nuclei purification from root tips. Those nuclei were successfully used to performed ATAC-seq experiments that revealed open chromatin regions in the wheat genome that will be useful to identify cis-regulatory elements. The INTACT system presented here will facilitate the development of ATAC-seq datasets in other tissues, growth stages, and under different growing conditions to generate a more complete landscape of the accessible DNA regions in the wheat genome.

**Supplementary Information:**

The online version contains supplementary material available at 10.1186/s12870-023-04281-0.

## Background

During the last 20 years, the significant progress in DNA sequencing technologies has allowed the rapid sequencing of complete genomes from multiple species, including a large number of varieties within each species. Today we have good reference genomes and pangenomes for the principal crop species, and that information has been critical to understand the genetic basis of biological processes, to identify natural variation responsible for phenotypic diversity, and for other applications.

A critical aspect of this information is the annotation of genes and regulatory sequences that control their expression. Currently, it is easier to identify and annotate the gene repertory present in a genome than their *cis*-regulatory elements and modules, which represents an important gap in our understanding of genome functionality [reviewed in [1]]. The relevance of these regulatory elements is highlighted by their significant enrichment at intergenic QTL in the maize (*Zea mays* L.) genome [2, 3].

Epigenome maps have contributed to the identification of regulatory regions, which are characterized by specific chromatin marks or signatures [1–6]. Another characteristic of these regulatory regions is their higher DNA accessibility. The fundamental units of chromatin are the nucleosomes, which consist of ~ 150 bp of double-stranded DNA wound around an octameric histone protein core connected by approximately 50 bp of linker DNA. The density of nucleosomes varies across the genome, with transcriptionally inactive heterochromatic regions characterized by high nucleosome occupancy and low turnover rate, and transcriptionally active regions showing low nucleosome occupancy and high turnover rate. The latter are frequent immediately upstream of transcription start site regions resulting in higher DNA accessibility [6, 7].

Regulatory elements are frequently located in regions of open chromatin, that is free of nucleosomes or are under dynamic nucleosome turnover, and those regions might act as platforms for recruiting both positive- and negative-acting transcription factors [1, 6, 7]. Therefore, different approaches have been developed to identify accessible chromatin regions to predict potential regulatory elements. Nuclease sensitivity, which was one of the first approaches, involves treating the cell nuclei with different concentrations of DNA nucleases and subsequently sequencing the small (< 500 bp) fragments of genomic DNA flanked by nuclease cleavage sites. Open chromatin regions are hypersensitive to nuclease digestion and are preferentially recovered under light-digestion conditions. DNase I hypersensitivity assays have been used to identify open chromatin regions in rice (*Oryza sativa* L.), maize, and *Arabidopsis thaliana* (L.) Heynh [4, 8–11]. A similar approach that uses micrococcal nuclease (MNase-seq) has been used to identify open chromatin regions in the Arabidopsis, maize, rice, and wheat (*Triticum aestivum* L.) genomes [2, 12–15].

An alternative strategy to assess chromatin structure uses an engineered Tn5 transposase (TN5) that simultaneously cleaves accessible DNA and integrates sequencing DNA adaptors [assay for transposase-accessible chromatin = ATAC, [16]]. Those adaptors are used later in a PCR amplification to generate sequencing libraries. Compared to nuclease sensitivity methods, ATAC-seq libraries preparation is simpler and requires less cell nuclei, which has facilitated the characterization of open chromatin regions in multiple crop species. In recent years, ATAC-seq has been used to identify DNA regulatory regions in multiple angiosperms, including Arabidopsis, *Medicago* L., tomato (*Solanum lycopersicum* L.), rice, maize, sorghum (*Sorghum bicolor* (L.) Moench), barley (*Hordeum vulgare* L.) and wheat [3, 5, 17–22].

An important parameter to assess chromatin accessibility in all these methods is the quality of the cell nuclei isolations. Broken DNA generated during extraction reduces signal/noise ratio and reduces the ability to detect open chromatin regions, so it is critical to start these experiments with high quality chromatin [19]. For ATAC-seq experiments it is also important to purify nuclei from plastids and mitochondria organelles because crude nuclei extracts result in a large fraction of reads coming from organellar DNA [19, 20]. Therefore, developing protocols to isolate high quality nuclei is critical for ATAC-seq experiments, particularly for species with large genomes like wheat.

High quality ATAC-seq data was recently reported in wheat using nuclei isolated from leaf protoplast [21]. However, nuclei purification from protoplast is very laborious, and the results could contain changes in open chromatin regions introduced by the protoplast preparation and purification method. An alternative method to purify intact cell nuclei, named INTACT [isolation of nuclei tagged in specific cell types [23, 24]], has been successfully combined with ATAC-seq in Arabidopsis, rice, tomato and Medicago [20]. This approach involves generating two transgenic lines, one expressing a chimeric nuclear envelope targeting fusion protein (NTF) that includes a nuclear binding domain (WPP), a reporter gene and the biotin ligase recognition peptide (BLRP), and the other line expressing the biotinylating enzyme BirA [25]. After combining both transgenes in a single plant by crossing, BirA will biotinylate the chimeric NTF protein bound to the nuclear envelope, and intact nuclei can be purified using magnetic beads conjugated with streptavidin [23, 24]. The purified nuclei can then be used for different purposes, including ATAC reactions. Comparisons of ATAC-seq data generated from crude nuclei extracts and INTACT nuclei isolation in Arabidopsis have shown higher quality results from the latter [20].

In this work, we developed a transgenic INTACT system in tetraploid wheat (*Triticum turgidum* subsp. *durum* (Desf.) Husn.) and showed that it can be used to purify nuclei using a simple and rapid protocol. We then combined our INTACT system with an ATAC-seq protocol and tested different conditions to optimize enrichment of open chromatin regions. Finally, we generated INTACT-ATAC-seq data from wheat root tip samples from frozen and fresh material. Our root ATAC-seq results showed good reproducibility and enrichment of open chromatin regions in intergenic and promoter regions, which is expected for regulatory elements. A similar distribution of accessible DNA was observed when we re-analyzed published wheat ATAC-seq data generated from wheat leaf protoplast [21]. In addition, we observed that approximately 50% of the open chromatin regions in the leaf protoplast ATAC-seq data was shared with our root ATAC-seq, indicating a high reproducibility between the two experiments and a large fraction of open chromatin regions shared between root and leaf tissues. Finally, both ATAC-seq datasets contain regions of open chromatin that overlap with previously functionally validated cis-regulatory elements, indicating that these data will be useful to identified other functional accessible DNA regions in the wheat genome.

## Results and discussion

### A wheat INTACT system allows high-quality and rapid nuclei purifications from root tips

**Fig. 1:**
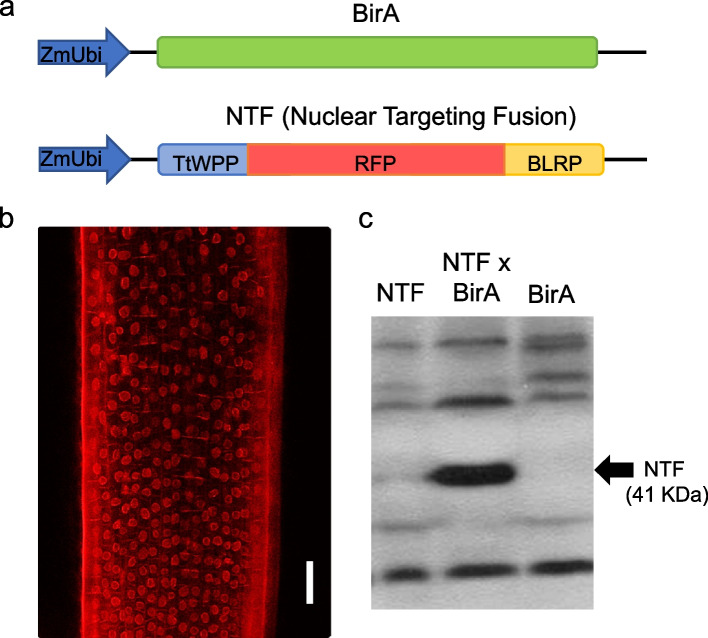
Development of INTACT system in tetraploid wheat Kronos. **a** Constructs used to generate transgenic plants expressing *E. coli* biotin ligase (BirA) gene and nuclear targeting fusion protein (NTF). The chimeric NTF protein consists of the WPP domain of wheat RanGAP1 for nuclear envelope targeting, RFP for visualization and the biotin ligase recognition peptide (BLRP), for biotinylation by BirA (full sequence in Fig. S1A). **b** RFP signal around nuclei in the roots of Kronos plants transformed with the NTF vector. Scale 100 µm. **c**. Western blot of proteins extracted from NTF and BirA lines and from their F_1_ hybrid. The biotinylated NTF protein was detected in the F_1_ hybrid but not in the individual transgenic plants (full gel in Fig. S1B)

To generate an INTACT system in wheat, we separately transformed its two components into the tetraploid wheat variety Kronos. To generate the line expressing the *Escherichia coli* biotin ligase (BirA) gene, we cloned the coding sequence of the BirA gene in a binary vector under the maize *UBIQUITIN* promoter (Fig. 1a) and used it to transform Kronos using *Agrobacterium-*mediated transformation. To generate the chimeric nuclear targeting fusion protein (NTF), we identified and cloned the region encoding the WPP nuclear binding domain from the wheat *RanGAP1* gene (*TraesCS3B02G433100*). The wheat WPP domain was then fused in frame with the reporter gene RFP (RED FLUORESCENT PROTEIN) and the biotin ligase recognition peptide (BLRP) (full sequence in Fig. S1), and this construct was also cloned in a binary vector under the maize *UBIQUITIN* promoter (Fig. 1a) and used to transform separate Kronos plants.

We first confirmed the expression of RFP around the nuclei in the roots of plants transformed with the NTF vector (henceforth NTF lines) using a confocal microscope (Fig. 1b). Next, we crossed the NTF lines with the transgenic plants expressing BirA (BirA lines) and confirmed NTF biotinylation in the F_1_ plants but not in the individual parental lines using Western blots (Fig. 1c). Together these results indicate that wheat NTF can localize to the nuclear envelope and be biotinylated by BirA. Since we use the constitutive maize *UBIQUITIN* promoter to drive the expression of both transgenes, this system results in the biotinylation of nuclei throughout the plant and, thus, is amenable to affinity purification with streptavidin beads from different tissues.

**Fig. 2:**
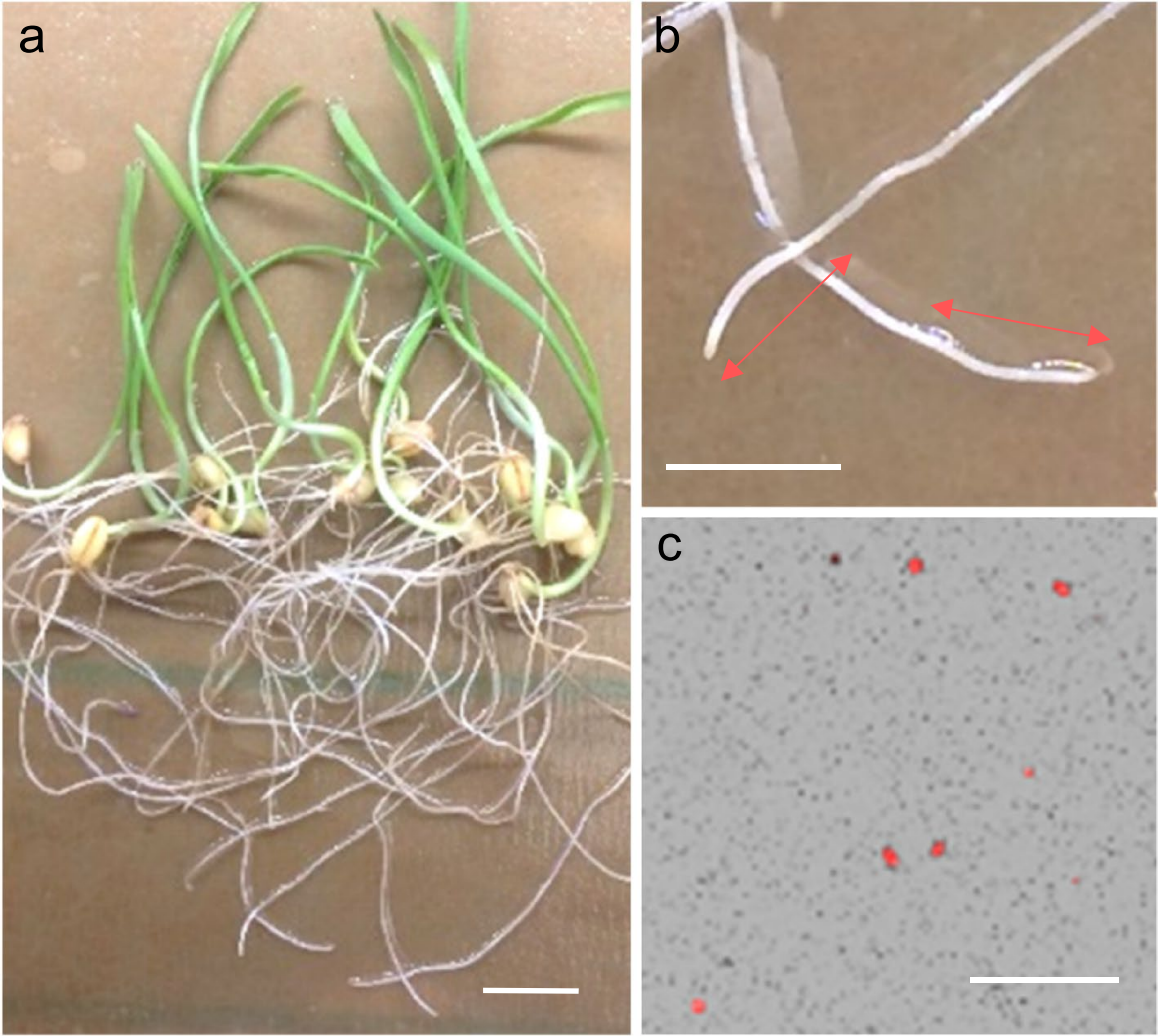
INTACT nuclei purification from wheat seedling root tips. **a** 7 days-old seedling used for INTACT nuclei extraction. Scale 1 cm. **b** Root tips collected, 1 cm long. Scale 1 cm. **c** INTACT-Purified nuclei (red dots). The small black dots in the background are magnetic beads. Scale 100 µm

Next, we tested the wheat INTACT system in a nuclei purification protocol (see methods). In these experiments, we used fresh root tips from 7-day-old Kronos lines harboring both BirA and NTF transgenes (henceforth INTACT lines, Fig. 2a). We collected the distal 1 cm region of the seminal roots (Fig. 2b) from approximately 10 plants and chopped them with a sharp razor. The extract was filtered, washed, and incubated with magnetic beads coated with streptavidin. After three washes, the resuspended bead-bound nuclei were stained with ethidium bromide, visualized and quantified using a hemocytometer in a fluorescent microscope (Fig. 2c). Using this INTACT system and protocol, we purified approximately 50,000 intact nuclei from 8–10 seedlings in a short time (90 min). Similar results were obtained when root tips were collected, flash-frozen and ground in liquid nitrogen before being processed for nuclei isolation.

### Wheat INTACT-ATAC from root tips

We then tested if the wheat INTACT-isolated nuclei could be used in ATAC-seq (see methods). To optimize the conditions for the ATAC reactions, we performed transposition reactions using different amounts of nuclei (from 20,000 to 2,000 nuclei). Other groups have observed that reducing the amount of input nuclei relative to a fixed amount of Tn5 enzyme helps to improve signal/noise ratio [26]. Reducing the number of nuclei, and thus the number of genomes, increases the number of enzyme molecules per nuclei, and this is expected to result in higher complexity libraries, a trend that was confirmed experimentally in Arabidopsis [26].

**Fig. 3:**
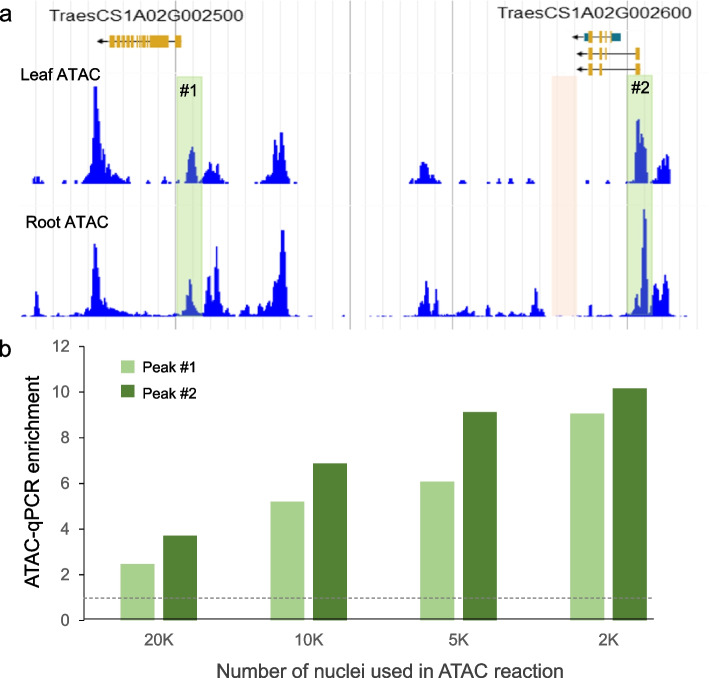
ATAC-enrichment in accessible *vs.* non-accessible DNA regions using different number of INTACT-nuclei. **a** Shot of a Chinese Spring (CS) genome browser showing previously published ATAC-seq data from wheat leaf protoplasts (top) [21] and ATAC-seq from fresh root tips generated in this work (bottom). Green rectangles including ATAC peaks #1 and #2 indicate accessible DNA, whereas the pink rectangle indicates non-accessible DNA. **b** ATAC-enrichment in peaks #1 and #2 relative to the non-accessible pink region was determined by qPCR using libraries prepared from different numbers of INTACT-purified nuclei from the fresh root tissue

We generated ATAC libraries using previously described protocols [18, 27]. To check the quality of the ATAC libraries we tested the enrichment of open chromatin regions selected from published ATAC-seq data generated from wheat leaf protoplast [21]. We designed primers covering two open chromatin regions and two close regions (Fig. 3a), and used them to check enrichment in the INTACT-ATAC libraries by qPCR (Fig. 3b).

We observed enrichment for the two open regions in all the ATAC reactions using different nuclei amounts, however the enrichment was clearly larger when we used a smaller number of nuclei (5,000 and 2,000) in the ATAC reactions (Fig. 3b), in agreement with results observed in Arabidopsis [26]. Therefore, we performed high-throughput sequencing of the ATAC-seq libraries generated using 5,000 to 2,000 nuclei. Note that the optimal nuclei number may depend on the tissue, so we recommend checking the enrichment of open regions by qPCR before proceeding to sequencing.

### Wheat root INTACT-ATAC-seq shows enrichment for open chromatin regions in intergenic and promoter regions

We generated ATAC-seq data from INTACT nuclei isolated from fresh and frozen wheat root tip tissue. The number of read pairs, reads aligned to wheat and other sequencing statistics are summarized in Table S1. In both libraries we obtained a small percentage of reads mapped to wheat plastids (2.64–3.04%) and mitochondria (5.94–5.87%) sequences, which was similar to the results reported for Arabidopsis root tips in INTACT-ATAC-seq (~ 3.5% plastid and ~ 2% mitochondria), and lower than that observed in ATAC-seq from crude nuclei extractions (~ 25% plastid and ~ 22% mitochondria) [20]. The percentage of chloroplast DNA in the root data was also lower than the amount reported in the wheat leaf protoplast (22.14%) [21].

**Fig. 4:**
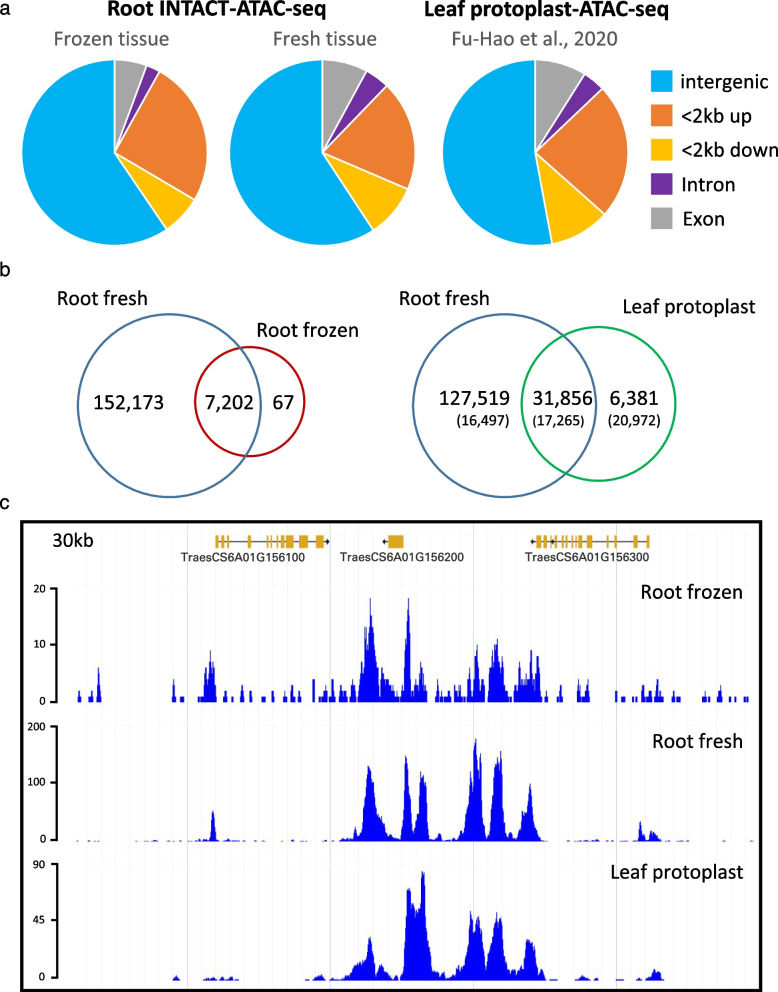
Comparison of ATAC-seq data from wheat root tips and leaf protoplast. **a** Genomic distributions of enriched regions (peaks) identified in INTACT-ATAC-seq generated from fresh and frozen root tips compared with those from leaf protoplast (including only high confidence annotated genes in Chinese Spring Ref Seq v1.1 from A + B + unassigned chromosomes). **b** Venn diagram showing the overlap between INTACT-ATAC-seq peaks observed in fresh and frozen roots tissue, and in published leaf protoplast. The numbers of peaks detected with 5.6 M reads in both leaf and root (down-sampling Figure S2) is indicated in parenthesis. **c** Shot of ATAC-seq data for a 30-kb region of the Chinese Spring wheat genome from INTACT-purified nuclei from frozen and fresh root tissue and reanalyzed published leaf protoplast data

We then called ATAC-seq peaks using the program MACS2 (max-gap 40 -q 0.01 [28]), and analyzed peak distribution along the wheat genome (Table S2). We divided the genome sequence in 5 categories: exons, introns, < 2 kb upstream of the 5’ end of the 5’UTR (< 2 kb up), < 2 kb downstream of the 3’ end of the 3’UTR (< 2 kb down), and intergenic space (> 2 kb upstream or downstream of genes) (Fig. 4a). In our two root datasets, we observed a similar distribution of peaks, which were mainly enriched in intergenic regions (59.5% frozen and 59.25% fresh), followed by regions within the 2 kb upstream (25.4% frozen and 19.2% fresh), the transcribed region of the gene regions including exons, introns, and 5’ and 3’ UTR sequences (8.1% frozen and 12.2% fresh), and the 2 kb region downstream of the 3’ UTR (7.0% frozen and 9. % fresh, Table S2, Fig. 4a). Similar genomic distribution of ATAC-seq peaks, with ~ 75% peaks outside transcribed regions, were previously reported in other angiosperms species, including Arabidopsis, *Medicago truncatula*, tomato, rice, maize, and barley [3, 5, 20]. Interestingly, Lu et al. (2019) observed that the distance of the open chromatin regions relative to the genes was larger in species with large genomes, with barley and maize having ~ 32.8% and ~ 45.9% of ATAC-seq peaks at > 2 kb from the genes. Those values are comparable to the proportion of peaks detected in the intergenic regions of the large wheat genome both in the root (~ 59%) and leaf protoplast datasets (54.3%. Table S2).

When we compared the ATAC-seq data generated from INTACT nuclei isolated from fresh and frozen root tissue, we observed that the fresh tissue generated a larger number of peaks (159,375 peaks) than the frozen tissue (7,269 peaks) (Fig. 4b, Table S2). Still, 99.08% of the peaks observed in the frozen root tissue overlapped (in at least 10% of their sequences) with the peaks from the fresh root material (Fig. 4b), indicating a good reproducibility of the INTACT-ATAC-seq to detect open chromatin regions in the wheat genome. The high reproducibility between these two experiments was also clear when the two datasets were visually compared in a genome browser (Fig. 4c). However, we noticed a higher background signal and a lower signal intensity in peaks from ATAC-seq data from the frozen tissue relative to the fresh tissue.

In agreement with the previous result, the SPOT score (for Signal Portion Of Tags), which is a measure of signal to noise ratio [29], was much lower for frozen ATAC-seq (0.006) than for fresh ATAC-seq (0.44) (Table S1), indicating a better signal/noise ratio in the latter. We speculate that the higher noise in frozen tissue libraries could be a result of the more aggressive grinding procedure, which may have resulted in a higher proportion of damaged chromatin. We also speculate that this could explain the reduced number of peaks called by MACS2 in the frozen root library, as it has been reported that the number of peaks called by MACS2 is strongly influenced by both sample quality and number of reads collected [26]. In agreement with this hypothesis, when peaks in frozen root were called using more relaxed parameters (max-gap 75 -q 0.05), we doubled the number of peaks (15,605) and still maintained a large overlap (94.48%) with fresh tissue (Table S3).

We then compared the root ATAC-seq distribution from Kronos with the published ATAC-seq data obtained from wheat leaf protoplast from the common wheat variety Paragon [21]. To this end, we re-analyzed the leaf protoplast ATAC-seq data using the same pipeline that we used for our root ATAC-seq data (Tables S1 and S2). Reads were first mapped to the CS RefSeq v1.0 reference, and then D genome results were eliminated for the comparison with the tetraploid genome (A and B genomes). Interestingly, the leaf ATAC-seq showed the same distribution of ATAC-seq peaks in intergenic and genic regions as the root tissue (Fig. 4a).

The leaf protoplast ATAC-seq generated fourfold fewer peaks (38,237 peaks with 5.6 M reads) than the fresh root (159,375 peaks with 59.3 M reads) due to its lower sequencing depth (Table S1). This explains the larger number of ATAC-seq peaks detected in the roots but not in the leaves (127,519, henceforth, RnoL peaks) than the ones detected in the leaves but not in the root (6,381, henceforth LnoR peaks, Fig. 4b).

To confirm that the difference in the number of peaks was caused by the differences in sequencing depth, we down-sampled the fresh root reads to 5.6 M reads as in the leaves (Fig. S2) and repeated the analysis. The down-sampling resulted in a large reduction in root peaks and a more similar number of LnoR and RnoL ATAC peaks (Fig. 4b numbers in parenthesis). However, as a result of the low coverage in both datasets, 14,591 peaks previously detected as overlapping peaks (henceforth L&R peaks) in the complete dataset were erroneously classified as LnoR (6,381 + 14,591 = 20,972 LnoR) in the reduced dataset.

The previous down-sampling result indicates that the classification of the leaf peaks based on the reduced dataset is less accurate than the one based on the complete fresh root dataset. Using the complete dataset, we found that 83.3% of the ATAC-seq peaks were detected in both the leaf protoplasts and the fresh root tips, and only 16.7% were detected only in the leaves (Fig. 4b, c). It should be noted that our root-leaf comparisons involve a tetraploid-hexaploid comparison (with eliminated D genome), and that sequences present in one of the genomes and absent in the other may generate differential peaks, contributing to an underestimation of the proportion of shared peaks between the two tissues. In spite of that, the large overlap between leaf and fresh root peaks indicates good reproducibility between the two ATAC experiments, and suggests that a large fraction of open chromatin regions is shared between roots and leaf protoplasts. This result is not surprising, since previous studies of open chromatin in different tissues from other plant species also observed large overlap between open chromatin regions from different tissues [2–4, 8, 10].

We then explored if the genes associated with the 6,381 LnoR peaks were preferentially expressed in the leaves relative to the genes associated with the 31,856 L&R peaks. For each peak, we identified the closest gene in each of the two classes, and then eliminated those present in both classes. This selects genes with all its associated peaks either in LnoR (LnoR-only) or L&R (L&R-only), generating two mutually exclusive categories. We then associated RNA-seq data for wheat leaf and root tissues [30] to each gene and classified them as enriched in leaf expression if their FPKM values in leaves were at least tenfold higher than in root and not enriched otherwise (Table S5). We performed a similar analysis for the RnoL peaks, but using the 16,497 root peaks identified in the down-sampling experiment, as we hypothesized that the full set of 127,519 peaks would contain a large fraction of false RnoL peaks due to the limited number of reads obtained in the leaf study (5.6 M).

We observed a significantly larger proportion (χ^2^ *P* < 0.0001) of genes with > tenfold higher expression in leaf than in root among those associated with LnoR-only ATAC peaks (20.4%) than among those associated with L&R-only peaks (11.2%, Table S5). Similarly, we observed a significantly larger proportion (χ^2^ *P* < 0.0001) of genes with > tenfold higher expression in root than in leaf among those associated with RnoL-only ATAC peaks (20.2%) than among those associated with L&R-only peaks (9.9%, Table S5). These results indicate that genes associated with ATAC peaks present only in one of the two tissues have a higher probability of being expressed preferentially in that tissue, although multiple other factors determine the final spatio-temporal expression of a particular gene.

### Normalized accessibility from root and leaf ATAC-seq correlates with gene expression

Chromatin accessibility upstream of the transcription start site (TSS) correlates positively with gene expression [31]. Therefore, we next analyzed the association between fresh root tips and leaf protoplast ATAC-seq and gene expression, using publicly available RNA-seq generated from root and leaf samples of hexaploid wheat [30].

**Fig. 5:**
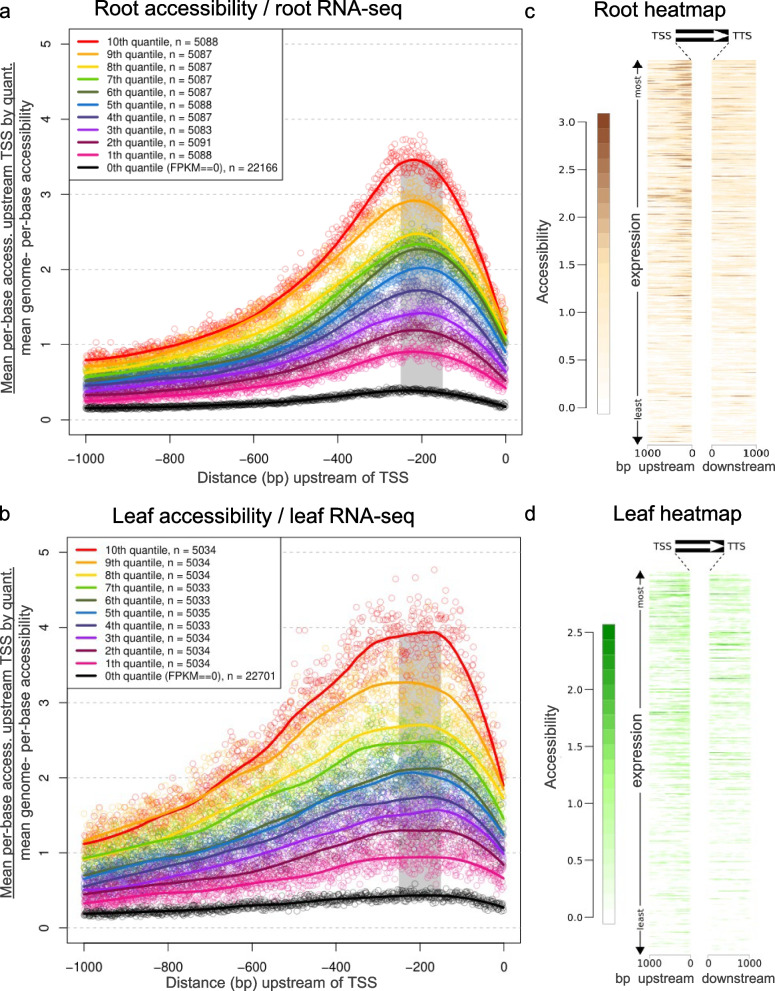
Normalized accessibility from root and leaf ATAC-seq correlates with gene expression. **a** and **b** Aggregate plots of per base TN5 insertion counts for the 1 kb region upstream of transcription start sites for high confidence genes in root (**a**) and leaf protoplast (**b**) ATAC-seq. Genes were stratified in 11 quantiles by expression level using previously published RNA-seq for root and leaf [30]. For each of the 11 stratified sets of genes, fold accessibility above genomic average was calculated for each of the 1000 bases upstream of transcription start sites, as indicated in the figure and described in the methods. A LOESS curve (Local Polynomial Regression) was added for dots corresponding to each gene set. Gray bars indicate a 150-bp region centered 225 bp upstream of the TSSs that was used to calculate average accessibility for each gene. **c** and **d** Heatmaps for accessibility 1 kb up- and down-stream of each gene for fresh root (**c**) and leaf protoplast (**d**) data. Genes are order from the highest expression on the top to the lowest expression in the bottom. The scale is log10 counts in 150 bp windows with 20 bp slide

To visualize the accessibility trends upstream of TSSs, we stratified expressed genes in ten quantiles (plus a non-expressed quantile) according to their expression levels in the different tissues (Fig. 5a and b). The data is also presented as heatmaps of the ATAC-seq signal (± 1 kb from each end) at all the annotated genes ranked by expression level (Fig. 5c and d).

As previously seen in Arabidopsis [30], in both wheat fresh root tips and leaf protoplast ATAC-seq we observed a peak of chromatin accessibility around 225 bp upstream the TSS of genes (Fig. 5a and b), with the most highly expressed genes (10^th^ quantile, red line) having the most accessibility and the other quantiles decreasing monotonically with expression (Fig. 5a and b). Heatmaps also showed higher chromatin accessibility closer to the TSS site of genes, which correlate with expression level (Fig. 5c and d). Supporting these observations, the Pearson correlation coefficient between total accessibility in a 150-bp window centered 225 bp upstream of the TSSs (indicated by gray bars in Fig. 5a and b) and gene expression was highly significant for both root and leaf (root: *R* = 0.364, *P* < 2.2e-16; leaf: *R* = 0.334, *P* < 2.2e-16). Therefore, as with other species, wheat ATAC-seq signal immediately upstream of gene transcription start sites is correlated to transcriptional activation, with other factors determining the final expression levels of particular genes in particular tissues or conditions. Heatmaps, also showed that 1-kb regions downstream of the transcription termination site (TTS), contain ATAC-seq peaks but at a lower frequency than in the upstream gene regions (Fig. 5c and d).

### Wheat ATAC-seq data overlap with putative regulatory elements

**Fig. 6:**
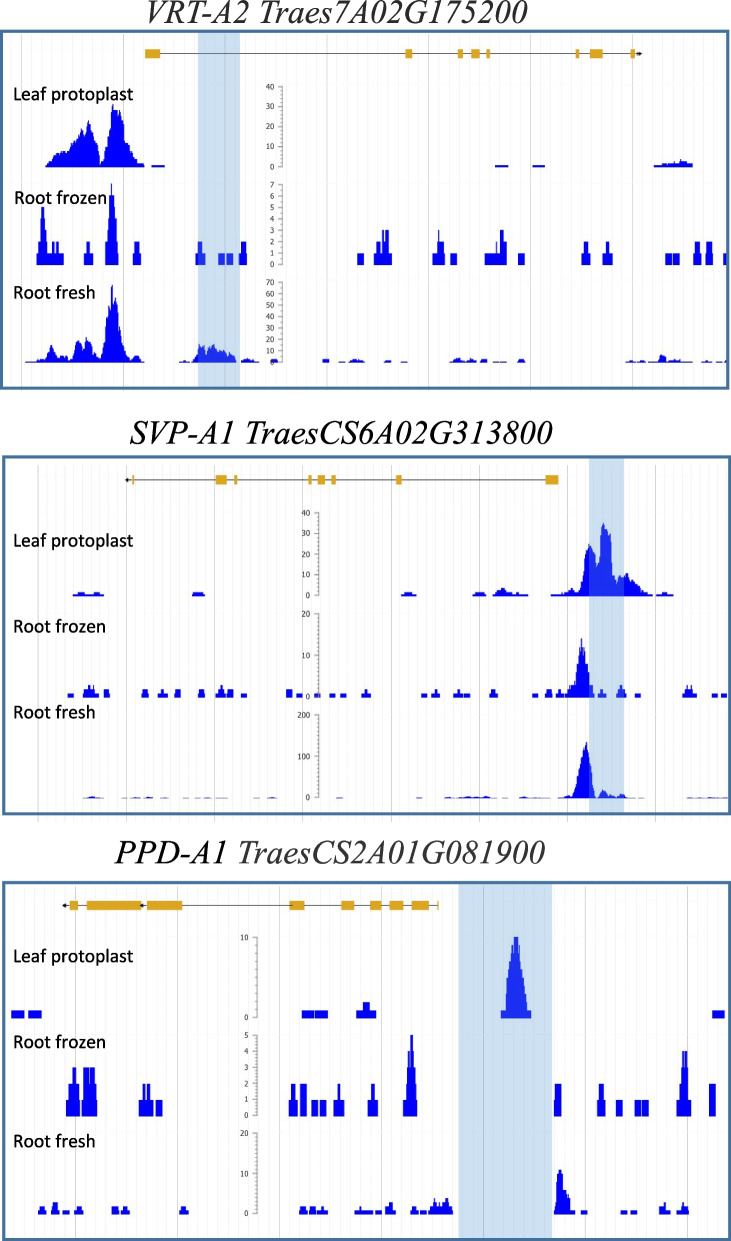
Wheat ATAC-seq data overlap with putative regulatory elements. Shot of a Chinese Spring genome browser ATAC-seq data showing putative regulatory elements in three wheat genes *VRT-A2*, *SVP-A1* and *PPD-A1*. Blue rectangles highlight the genetically mapped regulatory regions. Note that since Kronos has a 1027-bp deletion in *PPD-1A* promoter, no signal is detected in that region in both root ATAC-seq datasets

Finally, we used the ATAC-seq data to investigate the chromatin structure in putative regulatory elements previously identified in wheat by map-based cloning approaches (Fig. 6). Among them, we checked two loci, named *P1* and *P2*, that control glumes, lemmas, and grain length that were recently cloned and showed to encode a MADS-box transcription factor belonging to the *SHORT VEGETATIVE PHASE* family [32–34]. The *P1* and *P2* alleles were found in the tetraploid subspecies *T. polonicum* L. and *T. ispahanicum* Heslot, respectively, which are characterized by elongated glumes and grains [32–34]. The causal *P1* mutation is a sequence rearrangement in the first intron of *VRT-A2* in the *T. polonicum* allele, and in the case of *P2* a 482-bp deletion in the promoter of *SVP-A1* in the *T. ispahanicum* allele. In both cases, the mutations are associated with ectopic and higher expression of *SVP* alleles in the elongated glumes and floral organs, suggesting that they likely modify regulatory elements. Interestingly, we observed that both mutations overlap with ATAC-seq peaks in at least one of the datasets.

Variation in photoperiod response in wheat have been mapped to deletions in the promoter regions of the *PPD-D1* and *PPD-A1* homeologs [35, 36]. These mutations result in increased *PPD1* expression and accelerated heading under short day (SD) relative to the ancestral *PPD1* allele [35, 36]. The tetraploid wheat variety Kronos used in this study has a reduced photoperiod response conferred by the *Ppd-A1a* allele, which carries a 1,027 bp deletion in the promoter. Instead, the leaf protoplast ATAC-seq data was generated from the photoperiod sensitive hexaploid spring wheat variety ‘Paragon’. Therefore, we were able to check the promoter region of *PPD-A1* homeolog in both photoperiod insensitive and sensitive backgrounds. Interestingly, we observed a peak in the leaf protoplast ATAC-seq that overlaps with the region in Paragon that is deleted in Kronos. It was recently shown by ChIP PCR that the Evening Complex binds to this region repressing *PPD1* expression [37].

Taken together, the root and leaf ATAC-seq data overlapped well with putative regulatory elements previously identified by genetic mapping studies, and therefore can be a useful resource to identify regulatory regions in other wheat genes.

## Conclusions and future directions

We have developed and validated an INTACT system in tetraploid wheat that allows easy, rapid and high-quality nuclei purification from root tips. The current INTACT version uses the constitutive maize *UBIQUITIN* promoter and was designed to isolate nuclei from any tissue and developmental stages. However, this system can also be adapted to purify tissue-specific nuclei using tissue-specific promoters to drive the expression of the NTF construct [11, 20, 23, 24]. In addition to using the INTACT system in combination with ATAC-seq, we also envision the possibility of combining it with other methods for high-resolution mapping of chromatin features and transcription factor binding sites, such as the recently developed technology CUT&Tag [38].

We optimized the INTACT system in combination with ATAC-seq to identify open chromatin in wheat root tip samples. Multiple lines of evidence indicate that the root ATAC-seq results generated here are of good quality. First, we observed high reproducibility between two independent root experiments. Second, open chromatin regions were enriched in intergenic and promoter regions, which is expected for regulatory elements, and similar to the distribution of accessible DNA observed in other ATAC-seq studies [3, 5, 20, 21]. Third, we observed that 83% of the ATAC-seq peaks in the leaf protoplast ATAC-seq data were shared with our root ATAC-seq. Fourth, chromatin accessibility around the transcription start of genes significantly correlates with gene expression. Finally, we identify regions of open chromatin that overlap with previously functionally validated cis-regulatory elements in wheat.

The open chromatin regions identified in this study have been recently used in combination with 2-kb regions upstream of the 110,790 high-confidence wheat genes annotated in Chinese Spring (CS) RefSeq v1.0 to develop a second-generation capture panel for cost-effective sequencing of genome regulatory regions in wheat, which is commercially available from Arbor Biosciences [39].

Recent studies have shown that combining open chromatin maps with genome wide association studies data can reveal genetic variation in accessible regions significantly associated with quantitative traits [2, 3, 11]. Similarly, our ATAC-seq results would help wheat researchers to explore natural variation in regulatory regions as candidates for causal mutations associated with quantitative traits. Finally, the open chromatin regions identified here provide a useful resource to those interested in engineering existing regulatory networks to optimize plant growth and development by gene editing or TILLING approaches.

## Methods

### Plant material and growing conditions

In this study we used the tetraploid wheat variety Kronos (*Triticum turgidum* subsp. *durum* cv. Kronos), an old durum wheat cultivar released in 1993 that is publicly available from the US National Small Grain Collection (accession number PI 576,168). For all experiments, grains were first cold imbibed for 2–4 days at 4˚C in germination paper soaked with tap water, and then transferred to room temperature (21˚C) where they germinated. For the INTACT-ATAC experiments, root tips were collected from seedling grew under those conditions for 7 days (Fig. 2a). To advance generations and increase grain number, germinated transgenic seedlings were transferred to soil and grown in PGR15 growth chambers (Conviron) adjusted to 16 h of light (22 °C) and 8 h of darkness (18 °C). Intensity of the sodium halide lights measured at plant head height was ∼260 µM m^−2^ s^−1^.

### Vectors

To express the *E. coli* biotin ligase (BirA) gene, we PCR amplified the coding sequence of the BirA gene from a ACT2::Bir A vector [23] (primers listed in Table S4) and cloned it into pDONR vector (Invitrogen). We then subcloned it by L/R gateway reaction into the binary vector pLC41 (Japan Tobacco, Tokyo, Japan) downstream of the maize *UBIQUITIN* promoter.

To generate the NTF construct we used as template an Arabidopsis UBQ10::NTF vector where the original NTF construct (WPP-GFP-BLRP) was replaced by WPP-RFP-BLRP. We used the WPP domain of Arabidopsis RAN GTPase activating protein 1 (RanGAP1, locus At3g63130; amino acids 1–111, inclusive), which is necessary and sufficient for nuclear envelope association in plants, to identify the wheat homologues using blast. We amplified by PCR the WPP domain from the wheat RanGAP1 homolog (*TraesCS3B02G433100*) and used it to replace the AtWPP domain in the Arabidopsis WPP-RFP-BLRP construct by overlapping PCR (primers listed in Table S4). The final product was cloned in pDONR (Invitrogen), and then subcloned in pLC41. The vectors generated in this study will be available through ADDGENE.

### Western blot

Total protein was extracted from fresh leaves of 10-days-old plants. Leaves were ground in extraction buffer (50 mM Tris (pH8), 150 mM NaCl, 0.1% NP-40 with EDTA-free protease inhibitor) at tissue weight/buffer volume ratios of 1:1.5. Samples were separated by SDS-PAGE with 12% acrylamide and transferred to a nitrocellulose membrane. The membrane was blocked in PBSt (11.9 mM sodium phosphate, 137 mM NaCl, 2.7 mM KCl, 0.1% Triton X-100, pH = 7.4) with 10% milk for 30 min, washed twice for 5 min with PBSt, and incubated with a 1:2000 dilution of streptavidin-HRP (GE, catalog # RPN1231) in PBSt with 1% BSA for 30 min. The membrane was then washed three times for 5 min with PBSt and biotinylated proteins were detected using ECL detection reagents (Pierce, catalog # 34,075).

### Wheat transformation

Transgenic wheat plants were generated at the UC Davis Plant Transformation Facility (http://ucdptf.ucdavis.edu/) using the Japan Tobacco (JT) technology licensed to UC Davis. Immature embryos from Kronos were transformed using Agrobacterium EHA105. Selection of transgenic plants was conducted using hygromycin, and transgene insertion was validated by DNA extraction and PCR. The INTACT Kronos lines are available from the authors upon request.

### Wheat INTACT protocol

Our biotin labeled nuclei isolation protocol is an adaptation of a previous protocol from Queitsch Lab (http://www.plantregulome.org/media/protocols/data_assay/dnasei_arabidopsis/FINAL_whole_seedling_DNaseI_protocol.pdf). Briefly, root tips (1 cm long) from 8–10 7-day-old plants were collected in 1 mL of fresh ice-cold 1X Chopping Buffer (CB). To prepare 50 ml of fresh 1X CB, add 9.75 ml 5X CB [75 mM PIPES pH 6.5, 100 mM NaCl, 400 mM KCl, 5 mM EDTA pH 8.0, 1.5 M sucrose], 40 ml dH2O, 25 ul β-mercaptoethanol and 250ul 0.5 M spermidine, and keep on ice until used. After collection, the root tips were transferred to a weigh boat on ice containing 1 mL of ice-cold 1X CB, and chopped for 2.5 min using a sharp razor blade. The resulting extract was decanted into a 50 mL Falcon tube. Next, 2 mL of 1X CB were added to the samples and chopped again for 2 min. The extracts were combined to a final volume of ~ 4 mL, filtered through one layer of Miracloth and centrifuged in 50 mL tubes for 7 min at 1000 g at 4 °C. The nuclei pellets were carefully resuspended in 3 mL 1X CB using a wide-bore pipet tip to reduce mechanical shearing, and divided into two 2 mL-tubes (1.5 mL extract per tube). In parallel, 50 µL/per sample of Streptavidin Dynabeads (NEB S1420S) were pre-washed in 1X CB, of which 30 µL were added to each tube having the nuclei extract. The tubes with the bead-extract mix were placed in a rotator and incubated for 30 min at 4 °C with gentle rotation. Then, the tubes were incubated for 10 min in a magnet and the supernatant removed. Next, 1.8 mL of fresh ice-cold 1X CB + triton (to 50 ml of fresh 1X CB add 2.5 ml of 20% Triton X) were added to the tubes, which were rotated for 5 min at 4 °C, incubated in a magnet for 5 min and the supernatant removed. This procedure was repeated two more times, and finally the bead-bound nuclei were resuspended in 500 µL of 1X CB and placed on ice until transposition. Freshly purified nuclei in 1X CB to be used for ATAC-seq were kept on ice prior to the transposase integration reaction and never frozen.

### ATAC-seq

We selected root tips for our initial ATAC-seq experiments because INTACT was successfully used in root tips from multiple species before, including the monocot species rice [20]. These previous studies provided us important information to compare our results and troubleshoot the protocol. An additional motivation to select this tissue was the absence of wheat ATAC-seq data in roots. We tested a similar protocol in leaf tissue, but we observed a poor signal to noise ratio, suggesting a higher sensitivity of the leaf nuclei to rupture. Additional efforts will be required to optimize the conditions for leaf tissue.

Transposase integration reactions and sequencing library preparations were performed as previously described [18]. Briefly, 20,000 to 2,000 purified nuclei were used in each 25 µL transposase integration reaction for 30 min at 37 °C using Nextera reagents (Illumina; FC-121–1030). DNA fragments were purified using a column-based kit (DNA Clean & Concentrator™-5, Zymo Research) following manufacturer’s instructions and eluted in 15 µL elution buffer. Next, each sample (10 µl) was amplified using High Fidelity PCR Mix (NEB) and custom barcoded primers (Table S4) for 8 to 10 total PCR cycles. Each library (2 µL) was run on an agarose gel to visualize the average size of the amplified fragments. The amplified libraries were then purified using a column-based kit (DNA Clean & Concentrator™-5, Zymo Research) following manufacturer’s instructions, eluted in 15 µL elution buffer, quantified using qbit (Invitrogen) prior to pooling and sequencing. For ATAC enrichment check by qPCR, the amplified libraries were diluted 10 times in water, of which 5 μl were used in qPCR reaction with primer listed in Table S4. Quantitative PCR was performed using SYBR Green and a 7500 Fast Real-Time PCR system (Applied Biosystems).

### Sequencing and data analysis

Sequencing was performed using the Illumina NextSeq 500 or HiSeq 2000 instrument at the Genomics Facility at the University of Washington. Sequencing reads, 150 bp paired-end reads, were aligned to a genome consisting of the A and B genomes plus unassigned chromosome contigs of the hexaploid wheat sequence (RefSeq v1.0) [A + B + Unassigned], chloroplast (MH051715.1), and mitochondria (MH051716.1) genomes using bwa v0.7.15. Leaf protoplast ATAC-seq data [21] was generated in the hexaploid wheat cultivar Paragon. To compare the Paragon and Kronos ATAC-seq data, we downloaded and re-analyzed the Paragon leaf protoplast ATAC-seq data by aligning it to the combined CS RefSeq V1.0 genome, chloroplast (MH051715.1), and mitochondria (MH051716.1) genomes, and then subtracting the D genome results. We then generated per base bed files, containing the number of TN5 insertions per individual base (each pair of reads contributing two TN5 insertion sites), and density files for use in a genome browser (e.g., IGV). The density files we generated are sliding histograms of TN5 counts with bin size of 150 bp, slide of 20 bp, with each 150-bp window represented by a bar covering the center 20 bp of that window and the height representing the total number of TN5 insertions in the 150 bp window. ATAC-Seq peak regions were called using MACS2 (v2.2.7.1), -g 15e9 –max-gap 40 -q 0.01 [28]. ATAC-Seq peaks for which the distance between proximal ends was < 10 bp were merged.

Aggregate plots of per base TN5 insertion counts (Fig. 5) were generated for the 1 kb region upstream of transcription start sites for high confidence genes (iwgsc_refseqv1.0_HighConf_2017Mar13_parts.gff3).

To compare the relative accessibility and the expression level of the genes, we stratified genes by expression level using previously published RNA-seq for root at “First leaf through coleoptile” stage, and leaf at “3 leaves unfolded” stage [30]. FPKMs were determined by aligning raw reads to the hexaploid wheat sequence genome (RefSeq v1.0), chloroplast (MH051715.1), mitochondria (MH051716.1), and High Confidence annotated genes (iwgsc_refseqv1.0_HighConf_2017Mar13_parts.gtf)] using Tophat (v2.1.2) and Cufflinks (v2.2.1). Genes with an FPKM greater than zero were split into ten quantiles. Average per-base accessibility upstream of transcription start sites was determined for each of the 11 stratified sets of genes. We normalized each per-base value by dividing by the genome-wide per-base accessibility to determine the fold accessibility over background (Fig. 5). A LOESS curve (Local Polynomial Regression) was fitted to the dots corresponding to each gene set.

## Supplementary Information

**Additional file 1:** **Figure S1.** Validation of INTACT system in tetraploid wheat Kronos.

**Additional file 2:** **Figure S2.** Effect of the number of sequencing reads on the detected ATAC-seq peaks.

**Additional file 3:** **Table S1.** Sequencing stats.

**Additional file 4: Table S2.** Intact results and distribution in different genome regions.

**Additional file 5:** **Table S3.** Overlap between peaks from fresh and frozen roots ATAC-seq data.

**Additional file 6: Table S4.** Primers used in this study.

**Additional file 7:** **Table S5.** Expression of genes associated with ATAC seq peaks in different tissues.

## Data Availability

The datasets generated and/or analyzed during the current study are available in the NCBI repository as PRJNA878550 (www.ncbi.nlm.nih.gov/sra/?term=PRJNA878550, frozen root ATAC-seq) and PRJNA878551 (www.ncbi.nlm.nih.gov/sra/?term=PRJNA878551, fresh root ATAC-seq). The INTACT vectors and lines generated during the current study will be available from ADDGENE and from the corresponding author on request. Both the root and leaf ATAC-Seq data can be accessed through the GrainGenes Genome Browsers by selecting the CS reference genome RefSeq v1.0 (https://wheat.pw.usda.gov/GG3/genome_browser).

## Abbreviations

ATAC: Assay for transposase-accessible chromatin
BLRP: Biotin ligase recognition peptide
FPKM: Fragments per kilobase per million
INTACT: Isolation of nuclei tagged in specific cell types
NTF: Nuclear envelope targeting fusion protein
LOESS: Local polynomial regression
qPCR: Quantitative PCR
RFP: Red fluorescent protein
SPOT: Signal portion of tags
TN5: Tn5 transposase
TSS: Transcriptional start site
