# Supplementary material for: Optimization of ATAC-seq in wheat seedling roots using INTACT-isolated nuclei
Source: BMC Plant Biol. 2023 May 22;23:270. doi: 10.1186/s12870-023-04281-0 (PMC10201787; doi:10.1186/s12870-023-04281-0)

Figure S1. Validation of INTACT system in tetraploid wheat Kronos

a) Full sequence of the wheat chimeric nuclear targeting fusion protein (NTF).

>TtWPP-RFP-BLRP

ATGGCTTCAGCGGCGCCAGATTTCCAGCCCCGGACATTCTCCATCAAACTGTGGCCACCGAGTGAAAGCACTCGTCTGATGCTTGT
AGAGAGGATGACCAAGAACCTGTCCTCTGAGTCAATCTTTTCTCGCAAGTATGGCCTTTTGGGCAAAGAAGAGGCCCATAAGAATG
CCAAAAGGATTGAAGAGATGTGCTTTGCTTCTGCAGATGAGCATTTCAAGAAGGAGCCTGATGGTGATGGGAGTTCTGCTGTCCAG
CTATATGCAAAAGAAACAAGCAAGCTGATGCTGGAAGTCCTCAAAAAAGGTCCGGGGACGACTGCGGAACCAGAAGCGGCCGCTGC
CTCCTCCGAGGACGTCATCAAGGAGTTCATGCGCTTCAAGGTGCGCATGGAGGGCTCCGTGAACGGCCACGAGTTCGAGATCGAGG
GCGAGGGCGAGGGCCGCCCCTACGAGGGCACCCAGACCGCCAAGCTGAAGGTGACCAAGGGCGGCCCCCTGCCCTTCGCCTGGGAC
ATCCTGTCCCCTCAGTTCCAGTACGGCTCCAAGGCCTACGTGAAGCACCCCGCCGACATCCCCGACTACTTGAAGCTGTCCTTCCC
CGAGGGCTTCAAGTGGGAGCGCGTGATGAACTTCGAGGACGGCGGCGTGGTGACCGTGACCCAGGACTCCTCCCTGCAGGACGGCG
AGTTCATCTACAAGGTGAAGCTGCGCGGCACCAACTTCCCCTCCGACGGCCCCGTAATGCAGAAGAAGACCATGGGCTGGGAGGCC
TCCACCGAGCGGATGTACCCCGAGGACGGCGCCCTGAAGGGCGAGATCAAGATGAGGCTGAAGCTGAAGGACGGCGGCCACTACGA
CGCCGAGGTCAAGACCACCTACATGGCCAAGAAGCCCGTGCAGCTGCCCGGCGCCTACAAGACCGACATCAAGCTGGACATCACCT
CCCACAACGAGGACTACACCATCGTGGAACAGTACGAGCGCGCCGAGGGCCGCCACTCCACCGGCGCCTCCGGAGCTGCGGCCGCT
GCCATGGCTGGTGGACTTAACGATATCTTCGAAGCTCAGAAGATTGAATGGCATGAGGATACTGGTGGATCTTGA

b) Picture of the complete gel used in Figure 1. This gel includes multiple F_1 lines from crosses between Kronos BirA and NTF transgenic plants (both heterozygous for the transgene). Genotypes of the F_1 plants were determined by PCR and are indicated above each lane. Note that the biotinylated NTF protein (black arrows) is detected only in F_1 plants that have both transgenes. Figure 1 in the main text shows results within the dotted yellow square.

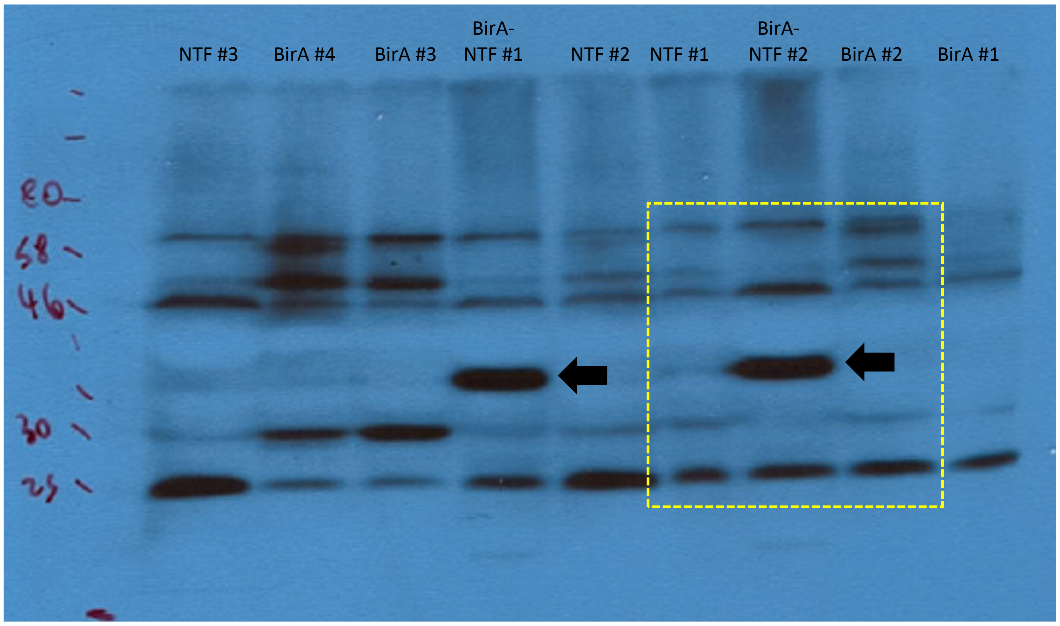

Supplement: Supplementary file 1 — Additional file 1: Figure S1. Validation of INTACT system in tetraploid wheat Kronos. [file 12870_2023_4281_MOESM1_ESM.pdf]
