# Supplementary material for: Optimization of ATAC-seq in wheat seedling roots using INTACT-isolated nuclei
Source: BMC Plant Biol. 2023 May 22;23:270. doi: 10.1186/s12870-023-04281-0 (PMC10201787; doi:10.1186/s12870-023-04281-0)

Figure S2. **Effect of the number of sequencing reads on the detected ATAC-seq peaks**

ATAC-seq peaks were determined using the program MACS2 [max-gap 40 -q 0.01] for both leaf (closed green diamond) and fresh root (open brown circles) data. Reads from leaves were analyzed with the full data (5.6 M reads, Table S1). The 59,336,265 reads from fresh roots were down-sampled at 5.6 M read (as in the leaves), and at 5.9 M read intervals (10% of total reads). The number of ATAC-seq peaks called on these various subsets (bam files) is plotted on the y-axis together with the number of peaks called on the 5.6 M leaf ATAC reads for comparison (green diamond). The number of peaks detected with 5.6 M reads is similar in the root and leaf data. The numbers of root peaks detected with the highest sub-sampling show the start of a plateau suggesting an adequate representation of the accessible chromatin in this tissue. By contrast, the number of ATAC peaks detected with the 5.6 M reads from the leaf protoplast ATAC-seq seems insufficient for an adequate representation of the open chromatin in these samples.

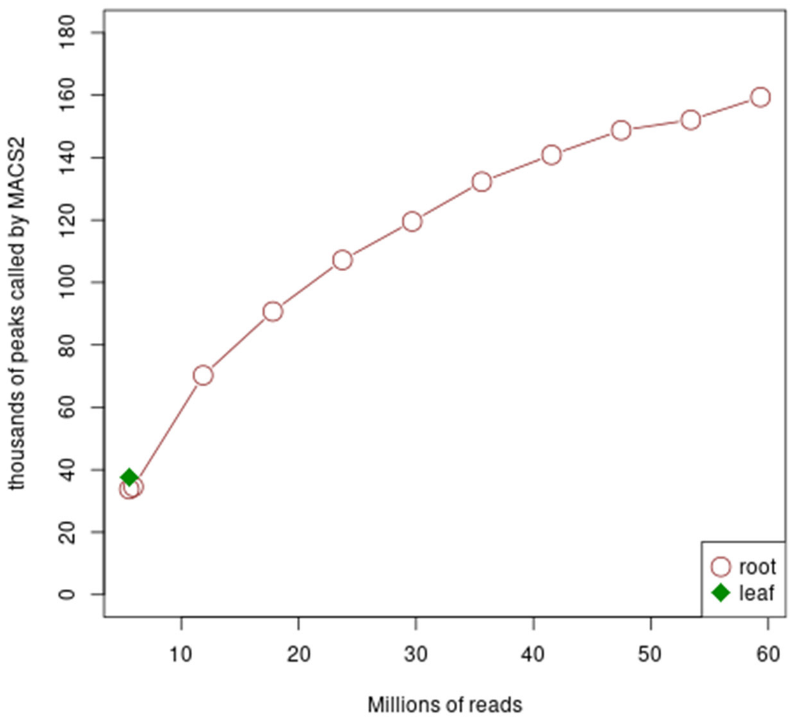

Supplement: Supplementary file 2 — Additional file 2: Figure S2. Effect of the number of sequencing reads on the detected ATAC-seq peaks. [file 12870_2023_4281_MOESM2_ESM.pdf]
